# Fully Organic Bulk Polymer with Metallic Thermal Conductivity and Tunable Thermal Pathways

**DOI:** 10.1002/advs.202004821

**Published:** 2021-05-24

**Authors:** Yongzheng Zhang, Chuxin Lei, Kai Wu, Qiang Fu

**Affiliations:** ^1^ College of Polymer Science and Engineering State Key Laboratory of Polymer Materials Engineering Sichuan University Chengdu 610065 P. R. China; ^2^ Key Laboratory for Soft Chemistry and Functional Materials of Ministry of Education Department of Polymer Science and Engineering School of Chemical Engineering Nanjing University of Science and Technology Nanjing 210094 P. R. China

**Keywords:** interfacial thermal resistance, organic bulk polymer, thermal conductivity, thermal management

## Abstract

Electrically insulating polymers are indispensable for electronic and energy applications, but their poor thermal conduction has increasingly become a bottleneck for high‐performance devices. Highly drawn low‐dimensional polymeric fibers and thin films can exhibit metallic conductivity. Extending this to bulk materials required by real world applications is prohibitive due to the additional interfacial thermal conduction barriers. It is demonstrated that highly aligned ultrahigh molecular weight polyethylene microfibers can be incorporated into a silicone matrix to yield a fully organic bulk polymer composite with a continuous vertical phonon pathway. This leads to a perpendicular thermal conductivity of 38.27 W m^−1^ K^−1^, at par with metals and two orders of magnitude higher than other bulk organic polymers. Taking further advantage of the mechanical flexibility of the microfibers, the processing method offers the freedom to tailor heat transfer pathways in a macroscopic 3D space. The material/process opens up opportunities for efficient thermal management in high‐performance devices.

## Graphical abstract

A versatile processing technique that allows achieving bulk processing of fully organic polymers into flexible forms with *κ* of 38.27 W m^−1^ K^−1^ is reported. This value is at par with some metals and two orders of magnitude higher than other bulk organic polymers. Moreover, the processing technique offers the unprecedented freedom to tailor heat‐transfer pathways in a macroscopic 3D space.

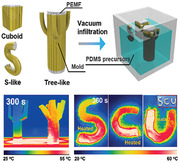

## 1 Introduction

Thermal management plays an increasingly important role in modern technologies, most notably electronics^[^ ^1^, ^2^, ^3^ ^]^ and energy devices.^[^ ^4^, ^5^, ^6^, ^7^ ^]^ The ease of processing and electrical insulation properties of organic polymers has made them indispensable for these applications.^[^ ^8^, ^9^ ^]^ Unfortunately, their typical poor thermal conduction has increasingly become a bottleneck toward designing powerful and highly efficient devices. Organic polymers typically exhibit thermal conductivity (*κ*) less than 0.4 W m^−1^ K^−1^, two orders of magnitude lower than metals (e.g., ≈36 W m^−1^ K^−1^ for steel). The poor *κ* of polymers originates from their disordered molecular configurations consisting of random coils and entanglements. Polymers with high structural order can exhibit high *κ*, including polyethylene fiber (20–104 W m^−1^ K^−1^),^[^ ^10^, ^11^ ^]^ polybenzobisoxazole fiber (≈60 W m^−1^ K^−1^),^[^ ^12^ ^]^ and nascent polyethylene film (≈60 W m^−1^ K^−1^).^[^ ^13^ ^]^ Harnessing this for real world applications requires a flexible processing technique to yield a bulk material without creating additional interfacial phonon scattering, which remains an unmet challenge. Judicious incorporation of high‐*κ* inorganic fillers (filler types and morphological manipulation) into a polymer matrix represents an alternative approach toward enhancing its *κ*. For instance, out‐of‐plane and in‐plane *κ* of 2.85 and 6.07 W m^−1^ K^−1^ have been realized for epoxy boron nitride nanocomposites.^[^ ^14^, ^15^ ^]^ Unfortunately, significant further enhancement is hindered by the inherently high interfacial thermal resistance (*R*) (10^−9^–10^−6^ m^2^ K W^−1^)^[^ ^14^, ^16^, ^17^ ^]^ in these multicomponent composites.

**Figure 1:**
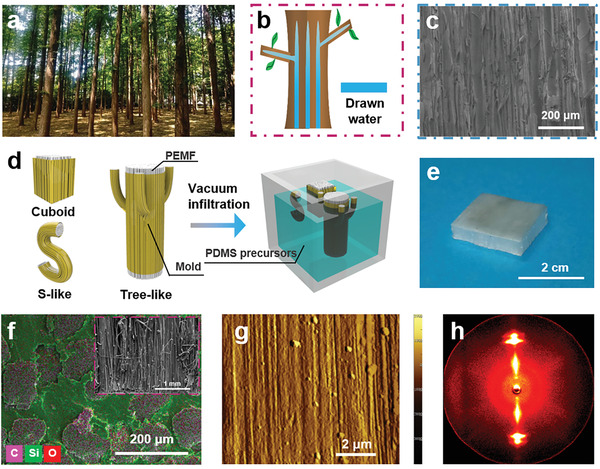
Design of PDMS/PEMF composites. a) Lush forest. b) Directional water transport in a towering tree. c) Scanning electron microscopy (SEM) image of the vertical xylem vessels in a tree. d) Fabrication of the PDMS/PEMF materials with multidirectionally aligned PEMF (PEMF‐A). e) Photo of the macroscopic PEMF‐A composite. f) SEM energy dispersive spectrum mapping image of PEMF‐A material from the horizontal cross‐section and SEM image on the vertical cross‐section direction (inset image). g) Atomic force microscope image of the etched PEMF. h) 2D wide‐angle X‐ray scattering image of the PEMF‐A material from the vertical cross‐section direction.

We take inspiration from trees to design a bulk processable polymer with high *κ* rivaling metals. Trees (**Figure** **1**a) can deliver water from the bottom all the way to the top owing to the microscopic xylem vessels parallel to the trunk and branches (Figure 1b,c). These microscopic and bottom‐up continuous vessels serve as straight‐through transporting highways that significantly enhance the water‐delivery efficiency.^[^ ^18^ ^]^ We hypothesize that constructing a similar phonon highway can lead to directional high *κ*, which puts forward two necessary requirements: i) directional alignment of thermal pathways; ii) bottom‐up continuous heat‐transfer paths without giving rise to additional interfacial phonon scattering.

## 2 Results and Discussion

Different from traditional thermoconductive fillers with micrometer‐size or nanosize dimension, which could not form continuous heat‐conduction paths within a long‐range distance (Figure S1, Supporting Information), we take advantage of highly stretched polyethylene microfibers (PEMF) with length more than several meters (Figure S2, Supporting Information) to guarantee the continuity of thermal pathways without bringing about additional interfacial phonon scattering. Detailed characterization of PEMF can be found in Figures S3–S5 in the Supporting Information. Figure 1d shows that these microfibers are first arranged into the multidirectionally aligned and continuous configuration with various complex shapes, i.e., cuboid, S‐like, or tree‐like shape, by a scalable mold‐fixation process. In particular, the shape and dimension can be tuned with an adjustable mold. This allows fine control of the packing density and content of PEMF. Polydimethylsiloxane (PDMS) liquid precursors are then vacuum infiltrated followed by its curing at 80 °C. The obtained bulk sample can be cut into desirable shapes using a room‐temperature water‐cutting technique, avoiding any localized heat to destroy the phonon‐delivery structure. With these structural processing procedures, PEMF is aligned in the PDMS matrix to yield a macroscopic bulk composite (i.e., the cuboid sample; Figure 1e). Scanning electron microscopic analyses (Figure 1f) confirm that vertically aligned PEMF wrapped around by PDMS run continuously across the entire vertical length of the composite. We call this composite PEMF‐A, in distinction to a PEMF‐R composite with randomly distributed PEMF. We note that crystallization in each individual PEMF forms fibrillar nanocrystals corresponding to extended chain fibrils with a diameter less than 100 nm (Figure 1g; Figure S6, Supporting Information), in contrast to the isotropically distributed crystals with randomly folded molecular conformation in the starting polyethylene powders. These fibrillar nanocrystals offer much higher axial phonon delivery than other crystalline types (i.e., spherulites, kebab, or shish‐kebab crystals).^[^ ^19^ ^]^ The favorable fibrillar nanocrystals, together with the microfiber alignment in PEMF‐A, create vertical phonon highways (Figure 1h; Figure S7, Supporting Information), enabling fast heat dissipation and a giant heat flux between the thermal interfaces (Figure S8, Supporting Information).

**Figure 2:**
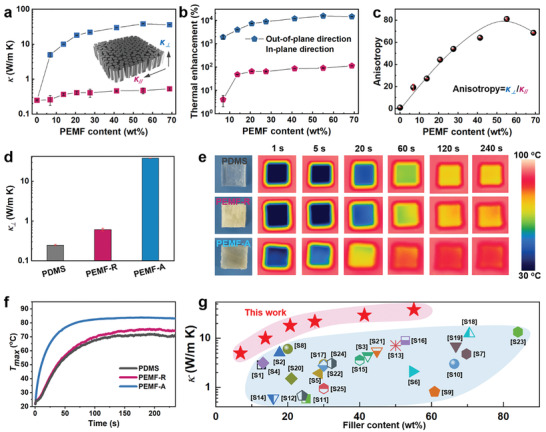
Thermally conductive characteristics of PDMS/PEMF composites. a) Anisotropic *κ*, b) thermal enhancement, and c) thermal anisotropy of PEMF‐A. d) Comparison between PEMF‐A, PDMS, and PEMF‐R. e) Infrared images and f) recorded temperatures in PDMS, PEMF‐R, and PEMF‐A as they are put on a hot plate set at 85 °C. g) Comparison between PEMF‐A and previously reported thermoconductive bulk polymer composites.

PEMF‐A exhibits strong anisotropic thermal conductivities (**Figure** **2**a,b). Its in‐plane thermal conductivity (*κ*_//_) is in the range of 0.24–0.53 W m^−1^ K^−1^, corresponding to horizontal thermal enhancement less than 112%. In stark contrast, the out‐of‐plane thermal conductivity (*κ*_⊥_) increases with the PEMF content, reaching 38.27 W m^−1^ K^−1^ at 55% PEMF content, corresponding to vertical thermal enhancement over 10 000%, and very close to the intrinsic *κ* of PEMF (≈63 W m^−1^ K^−1^, estimated according to Parallel Model in the Supporting Information). However, more than 55% addition of PEMF will result in small decrease of *κ*_⊥_ because of the difficult infiltration of PDMS matrix and thus some residual pores within the material. The thermal anisotropy (Figure 2c), defined as the ratio between *κ*_⊥_ and *κ*_//_, reaches a maximum value of 81. The *κ*_⊥_ of 38.27 W m^−1^ K^−1^ for PEMF‐A is 153 and 62 times higher than that of pure PDMS and PEMF‐R with an identical PEMF content (Figure 2d). This superior thermal performance of PEMF‐A is demonstrated as thermal interface materials (TIMs) in Figure 2e,f and Figure S10 in the Supporting Information, because of its much smaller bulk thermal resistance (i.e., 6.71 × 10^−5^ m^2^ K W^−1^ at the thickness of 2.5 mm) if compared with that of pristine PDMS (i.e., 1.04 × 10^−2^ m^2^ K W^−1^ at the thickness of 2.5 mm). Specifically, the above three samples are placed on a hot plate set at a constant temperature (65, 85, or 100 °C). PEMF‐A not only is heated at a much higher speed but also reaches a much higher ultimate temperature comparable to the hot plate. This phenomenon reflects its best thermally conductive characteristic among these three samples, enabling the highest heat flux in the thickness direction. Overall, the high *κ*_⊥_ of our PEMF‐A compares quite favorably with reported examples of inorganic filler‐based bulk polymer composites despite its full organic nature (Figure 2g; Table S2, Supporting Information).

**Figure 3:**
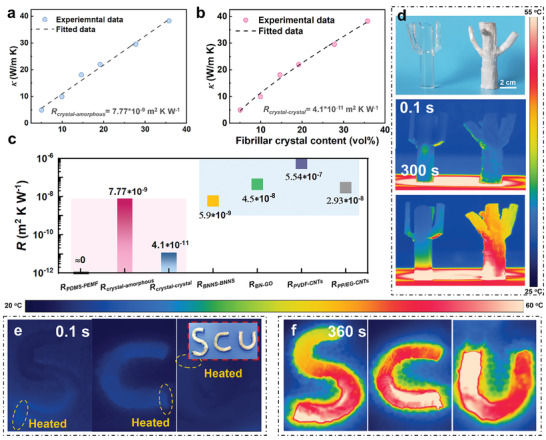
Interfacial thermal resistance and 3D tailorable heat transfer pathways in PEMF‐A composites. a) Experimentally measured *κ* and modified EMT model simulated *κ* of PEMF‐A material. b) Experimentally measured *κ* and Foygel model simulated *κ* of PEMF‐A material. c) Comparison of *R* values for PEMF‐A with those reported for polymer/filler composites. d) Optical images of PDMS (left) and PEMF‐A (right) materials with a tree‐like conformation, and their infrared images after different heating times on an 85 °C hotplate. e) Infrared images of PEMF‐A with “SCU” conformation (inset: optical image) before and f) after heating for 360 s.

We further analyze the internal interfacial phonon resistance (*R*) in order to understand the superior performance of PEMF‐A in the perpendicular direction. Here, *R* can arise from the acoustic or diffusion mismatch between the PDMS–PEMF interface, the crystal–crystal, and crystal–amorphous region interfaces within the PEMF bunches. Since PEMF is continuous in this direction, no additional PDMS–PEMF interfaces exist, namely, *R*_PDMS–PEMF_ ≈0 m^2^ K W^−1^. Using the modified effective medium theory (EMT) model,^[^ ^16^, ^20^ ^]^ the intrinsic *κ* of PEMF crystal according first‐principles calculation,^[^ ^21^ ^]^ the estimated *κ* of amorphous region of PEMF based on Series Model analysis (Supporting Information),^[^ ^13^ ^]^ and the dimension of fibrillar nanocrystals from the small angle X‐ray scattering analysis (Figure S11, Supporting Information), *R*_crystal–amorphous_ is quantified as 7.77 × 10^−9^ m^2^ K W^−1^ (**Figure** **3**a). A nonlinear model by Foygel et al.^[^ ^22^ ^]^ is used to fit (Figure 3b; Figure S11, Supporting Information) the interfacial contact resistance (*R*_crystal–crystal_), which is determined to be ≈4.1 × 10^−11^ m^2^ K W^−1^. These *R* values for our PEMF‐A, especially for *R*_crystal–crystal_ and *R*_PDMS–PEMF_ (Figure 3c), are orders of magnitude smaller than those reported for polymer/filler composite systems (10^−9^–10^−6^ m^2^ K W^−1^).^[^ ^14^, ^17^, ^20^, ^23^ ^]^ These synergistic hierarchical orders (fibrial crystals, bridge effect of amorphous region, and continuous macroscopic alignment) in our PEMF‐A create phonon highways responsible for its exceedingly high *κ*_⊥_.

The flexibility/deformability of the PEMF bunches allows harnessing the superior *κ* for manipulating thermal pathways in a macroscopic 3D space. Figure 3d shows a tree‐like PEMF‐A, along with a PDMS for comparison. Upon heating from the bottom, the heat is rapidly transported to the up (≈8 cm) and the branches for the PEMF‐A, in stark contrast to the PDMS reference. The thermal pathway can also be manipulated horizontally. In Figure 3e and Figure S12 in the Supporting Information, PEMF‐A processed as SCU letters are locally heated with a laser with the heating spots labeled. Remarkably, the localized heat is transported along the pathway defined by the letters (Figure 3f), with minimum dissipation to the surroundings. A video capturing this process is provided as Movie S1 in the Supporting Information.

**Figure 4:**
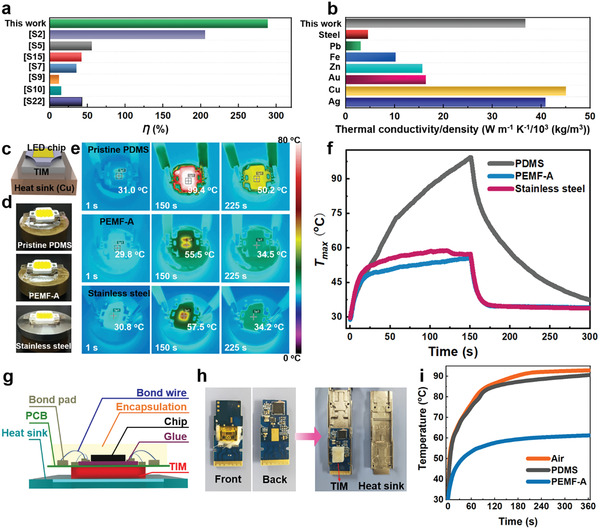
PEMF‐A for interfacial thermal management. a) Comparisons of *η* and b) thermal conductivity/density with other bulk materials. c) Schematic diagram, d) optical pictures, e) infrared images, and f) working temperatures for PEMF‐A or comparative materials as TIMs for thermal management of LED chips. g) Schematic diagram, h) optical pictures, and i) working node temperatures for PEMF‐A or comparative materials as a TIM for thermal management of a COB device.

The PEMF‐A exhibits the highest thermal conductivity enhancement per unit filler (*η* = 289%) when compared to other reported bulk polymer composites (**Figure** **4**a; Table S2, Supporting Information). In addition, given its fully organic nature, its light weight advantage is maintained since high *κ* inorganic fillers typically have high densities. In fact, the thermal conductivity per unit density (36.76 W m^−1^ K^−1^/10^3^ kg cm^−3^) for PEMF‐A is even comparable to that of silver (40.86 W m^−1^ K^−1^/10^3^ kg cm^−3^) and copper (45.06 W m^−1^ K^−1^/10^3^ kg cm^−3^) and superior to many other metals (Figure 4b).

Generally, ultrastretched macromolecular chain configuration is metastable, which can be relaxed at sufficiently high temperatures due to higher mobility of the chain segments. To our satisfaction, our PDMS/PEMF material can maintain good thermal stability up to 100 °C (Figures S13 and S14, Supporting Information). More importantly, our fully organic bulk polymer is electrically insulating (Figure S15, Supporting Information) and possesses high dielectric stability (low dielectric constant of 5.28–3.82 and suppressed dielectric loss of 0.117–0.011 at 0.1–10^6^ Hz; Figure S16, Supporting Information).

The performance package offered by PEMF‐A makes it a highly attractive candidate for use as the next‐generation TIMs. We use an LED chip as an illustrative example, with a copper block as the heat sink, our PEMF‐A as the TIM, and PDMS or stainless steel for comparison (Figure 4c,d). Figure 4e,f shows that, with pristine PDMS as the TIM, the working temperature of the LED can rise to ≈100 °C. By comparison, with our PEMF‐A material or stainless steel, heating or cooling during LED's lightening or extinguishing is much faster and the equilibrium working temperature is reduced sharply to around 55 °C. The similar performance between PEMF‐A material or stainless steel is particularly noteworthy as it highlights directly the metallic *κ* of the former. The thermal conducting performance is further demonstrated with chips on board (COB) with the PEMF‐A material as the TIM (Figure 4g,h). For minimized thermal contact resistance dependent on not only the thermal resistance of the PDMS/PEMF bulk material, a commercial thermal grease was used to decrease the interfacial thermal resistance.^[^ ^24^, ^25^ ^]^ Remarkably, the temperature increase during the device operation is almost 30 °C lower for PEMF‐A than those for the reference samples (Figure 4d). This highly efficient thermal management is directly translated into stable device performance (Figures S17–S20, Supporting Information).

## 3 Conclusion

Our fully organic bulk PEMF‐A composite allows seamless integration of metallic *κ* with lightweight, electrical insulation and mechanical softness (Figure S21, Supporting Information) in an unprecedented package. The freedom to manipulate thermal pathways also creates unique opportunities for directing thermal transport in a 3D space. Combined, these two features offer an attractive solution for efficient thermal management demanded for increasingly high‐performance devices (e.g., high‐power, high‐frequency, and complex integration).

## Conflict of Interest

The authors declare no conflict of interest.

## Supporting information

Supporting Information

Supplemental Video 1

## Data Availability

Research data are not shared.
