# Supplementary material for: Fully Organic Bulk Polymer with Metallic Thermal Conductivity and Tunable Thermal Pathways
Source: Adv Sci (Weinh). 2021 May 24;8(14):2004821. doi: 10.1002/advs.202004821 (PMC8292902; doi:10.1002/advs.202004821)
Supplement: Supplementary file 1 — Supporting Information [file ADVS-8-2004821-s002.pdf]

© 2021 Wiley-VCH GmbH

ADVANCED SCIENCE
Open Access

Supporting Information

for *Adv. Sci.*, DOI: 10.1002/advs.202004821

Fully Organic Bulk Polymer with Metallic Thermal Conductivity and Tunable Thermal Pathways

Yongzheng Zhang, Chuxin Lei, Kai Wu, Qiang Fu**

Supporting Information

Fully Organic Bulk Polymer with Metallic Thermal Conductivity and Tunable Thermal Pathways

Yongzheng Zhang, Chuxin Lei, Kai Wu, Qiang Fu**

Y. Zhang, C. Lei, Dr. K. Wu, Prof. Q. Fu,
College of Polymer Science and Engineering, State Key Laboratory of Polymer Materials Engineering, Sichuan University, Sichuan University, Chengdu 610065, PR China
E-mail: kaiwu@scu.edu.cn; qiangfu@scu.edu.cn
Y. Zhang, Dr. K. Wu,
Key Laboratory for Soft Chemistry and Functional Materials of Ministry of Education, Department of Polymer Science and Engineering, School of Chemical Engineering, Nanjing University of Science and Technology, Nanjing 210094, PR China

1. Materials

PEMF (diameter of ≈15 μm) fabricated via a gel spinning process was purchased from Shenzhen Teli Chemical Fiber Co., Ltd. PDMS precursors (SYLGARD™ 184) were obtained from Dow Chemical.

2. Preparation of PDMS/PEMF composites

The continuous and flexible PEMF bunches with suitable weight contents (0-68.85 wt%) were firstly placed in a metal mold. They were tightened with the help of the tunability of the dimension of the mold, and thus fixed to be the designed shape, such as simple cuboid, tree-like shapes, complicated "SCU" shapes. The viscous PDMS precursors were vacuum infiltrated into the customized PEMF bunches and cured at 80 ^{o}C for at least 8 h to obtain PEMF-A, which was cut into desirable dimension/shape with a high-pressure water-cutting technique. PEMF-R material was prepared by mixing PDMS precursors and random PEMF in a mold followed by a similar thermal curing procedure.

3. Characterization

Field-emission scanning electron microscope (SEM) and its mapping characterization was conducted with a JSM-7500F machine by Jeol for morphological observations of PEMF and PDMS/PEMF materials. The extended nanocrystals in PEMF were verified by an atomic force microscope (AFM, 359 Marin Keys Bivd, Suite 20, USA). 2D wide-angle X-ray scattering (2D WAXS) characterization was performed at room temperature using a Bruker D8 Discover X-ray diffractometer equipped with a Vantec 500 detector to analyze the oriented information. Raman spectra were collected with Aramis machine made by HORIBA JOBIN YVON, in order to describe the molecular orientation and crystallinity of PEMF. Small angle X-ray scattering (SAXS) was carried out on the GeniX 3D beam delivery system, using a MP-Xeuss 2.0 (BRUKER AXS, Inc) with Cu Ka radiation (λ=0.154 nm).

Laser flash analysis (LFA, NETZSCH, Germany) technique was used to quantify the thermal diffusivity at variable temperatures, and the testing voltage and pulse width were set to be 250 V and 600 μs, respectively. The different samples were tailored into the dimension of 10 mm*10 mm*x mm, where x is in the range from 2.5 mm to 4.0 mm. Anisotropic thermal conductivities of PDMS and various PDMS/PEMF composites were calculated according the equation $\kappa = \alpha*\rho*C_p$, where κ, α, ρ and C_p respectively correspond to anisotropic thermal conductivity, thermal diffusivity, density and specific heat capacity. C_p of PDMS and PEMF were respectively measured by differential scanning calorimetry (DSC, TA) using sapphire as the standard.

Dielectric properties were measured by a broad-frequency dielectric spectrometer Concept 50 (Novocontrol, Germany) over the frequency range from 10^{-1} to 10^{6} Hz at room temperature.

To visualize the thermal management of various materials, infrared camera (FLIR-T600) was used to record the temperature distribution of the samples. For evaluating the thermal management capacity, the samples were coated by graphite to guarantee their similar surface optical properties, and then placed on the hot stage (65 ^{o}C, 85 ^{o}C or 100 ^{o}C). The surface temperatures were then recorded by infrared camera. For the demonstration in Figure S12, black graphite was coated onto the intended heating spot followed by laser light irradiation (LSR808H-FC-5W, LASEVER INC.). Again, videos were captured by the infrared camera.

The COB device was measured by the assembled equipment including high-precision power supply (Agilent E3640A), 30G random signal generator, BER tester (Keysight N4960A, N4951A), and high-speed sampling oscilloscope (Keysight DCA-X 86100D, 83484 and 86105D components). Because the large air gap (≈ 2.5 mm) exists between the heat source on the printed circuit board and the metal shell, our PDMS/PEMF was tailored into the suitable dimension. Before the test, a commercial thermal grease was coated at the bottom and up surface of PDMS/PEMF bulk sample, and therefore the interfacial thermal resistance with heat source or heat sink would be minimized.

Mechanical compression test was carried out on an Instron 5967 universal tester (USA) with a 1 mm min^{-1} stretching velocity at room temperature.

4. Statistical Analysis

Quantitative data were expressed in form of means ± standard deviation, as shown like the error bars in the figures. At least three samples and five different positions in per samples were conducted for testing the average thermal diffusivity of prepared materials. The information about sample size was given in the respective figure captions. The experimental data of Raman spectroscopy was normalized by the OriginPro 2018 software (OriginLab, MA, USA). The experimental data of SAXS was conducted by the Fit2D (ESRF).

5. Supplementary figures and tables

5.1 Characterizations of PEMF and PE powder

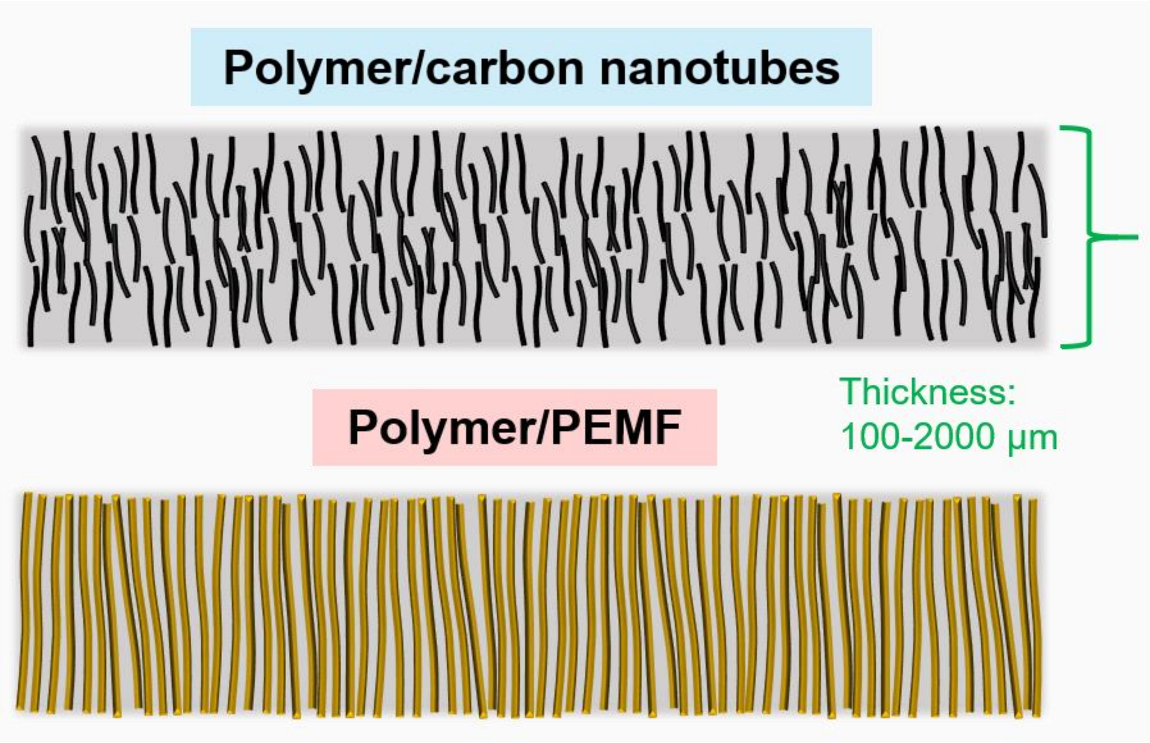

Figure S1. Schematic of cross-section morphology. Schematic of cross-section morphology of polymer/carbon nanotubes composites and polymer/PEMF material. Noted that PEMF could form the bottom-up continuous thermal pathways, while carbon nanotubes could not.

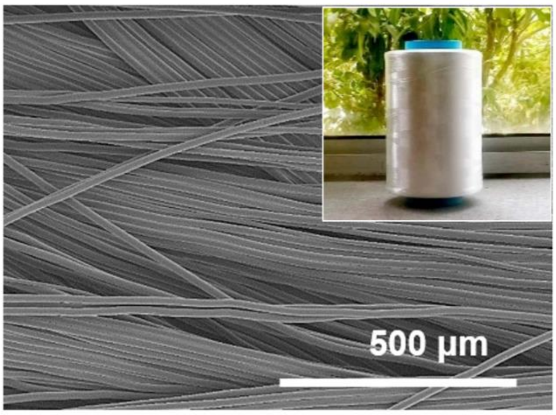

Figure S2. **SEM morphology of PEMF.** Inset picture shows the large-scale production and meter-scale length.

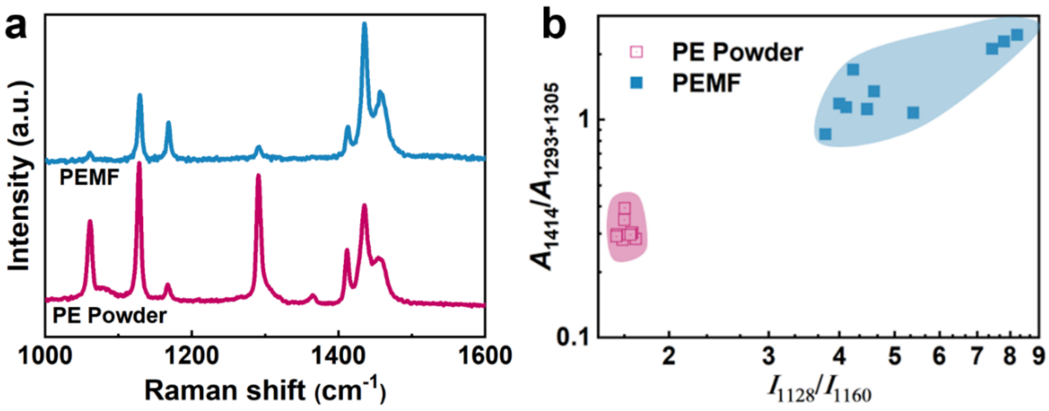

Figure S3. **Raman spectroscopy test.** (a) Raman spectra of polyethylene (PE) powder and PEMF, (b) and the statistical I_{1128}/I_{1160} vs $A_{1414}/A_{1293+1305}$ results according to the Raman spectra.

Raman spectra were used to analyse the molecular orientation and crystallinity (χ_c) of PE powder and PEMF, according to the peak intensity ratio of 1128 cm^{-1} and 1160 cm^{-1} (I_{1128}/I_{1160}), and the ratio of integral intensity areas of 1414 cm^{-1} to 1293 cm^{-1} and 1305 cm^{-1} ($A_{1414}/A_{1293+1305}$), respectively. It indicates that PEMF is provided with much higher molecular orientation after stretching due to a significant increase of I_{1128}/I_{1160}, at the same time that it also possesses higher χ_c than PE powder.

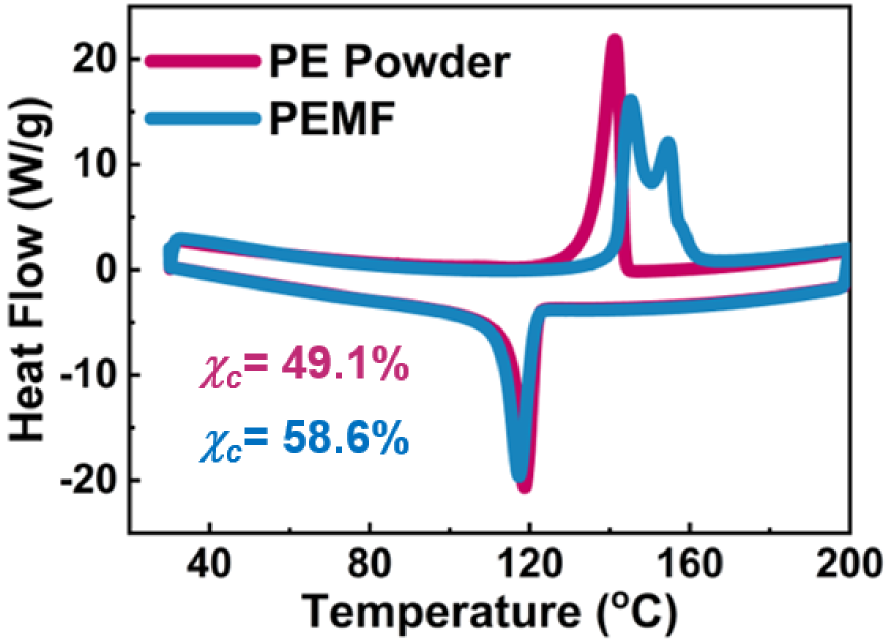

Figure S4. **DSC test.** DSC curves of raw PE powder and PEMF for further calculation of degree of crystallization.

The DSC results could also be utilized to calculate the χ_c of PE powder and PEMF. From the formula $\chi_c = \Delta H/\Delta H_c$, ΔH and ΔH_c correspond to melting enthalpy of chosen materials and melting enthalpy of PE with complete crystallizations, respectively. ΔH could also be gained by integrating the areas of peaks from the image. It is commonly acknowledged that the ΔH_c of PE material is 293 J/g as usual. According to DSC results, the PEMF (58.6%) behave higher χ_c than pure PE powder (49.1%), which is in accordance with the mentioned Raman spectra results.

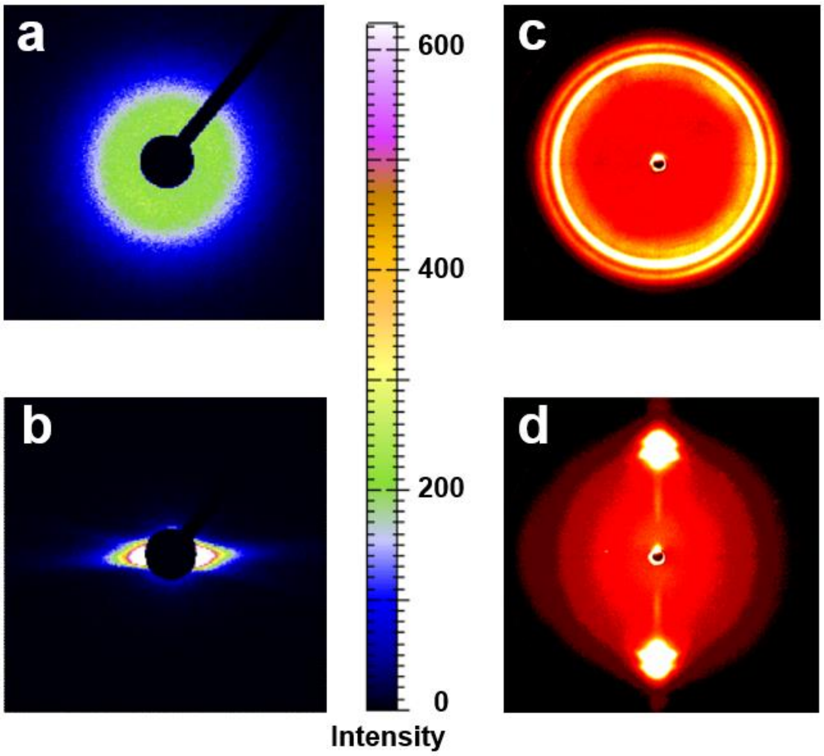

Figure S5. **SAXS and 2D WAXS characterizations.** SAXS images and of (a) PE powder and (b) PEMF. 2D WAXS images of (c) PE powder and (d) PEMF.

From ring diffraction pattern of Figure S5a to oriented diffraction pattern of Figure S5b, SAXS images demonstrate that isotropically distributed crystals in the powders with randomly folded molecular conformation has even been engineered into the fibrillar nanocrystals, with sufficient crystalline orientation in the axial direction of PEMF. Meanwhile, the two-point scattering patterns of Figure S5d also strongly prove the mentioned conclusion.

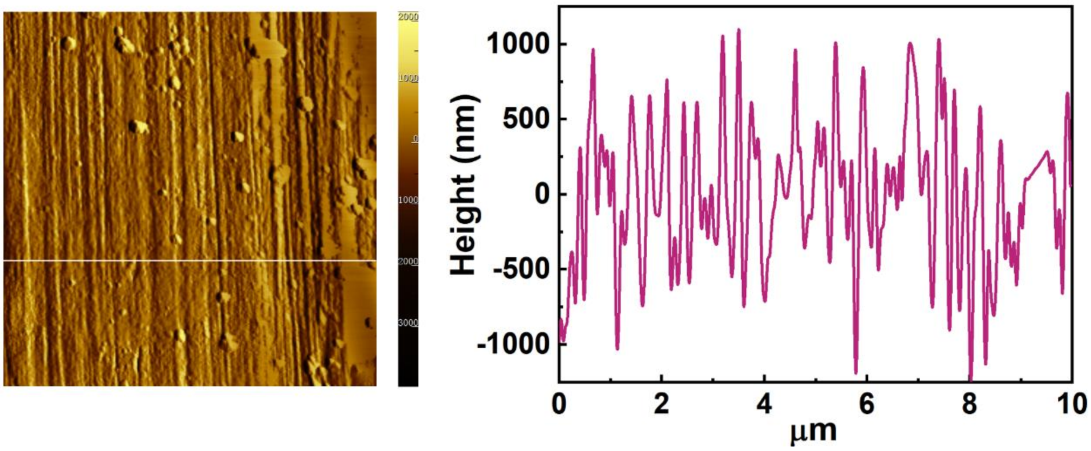

Figure S6. **AFM image of the etched PEMF and its corresponding height information.**

5.2 Morphological and thermal characterizations of PDMS/PEMF composites

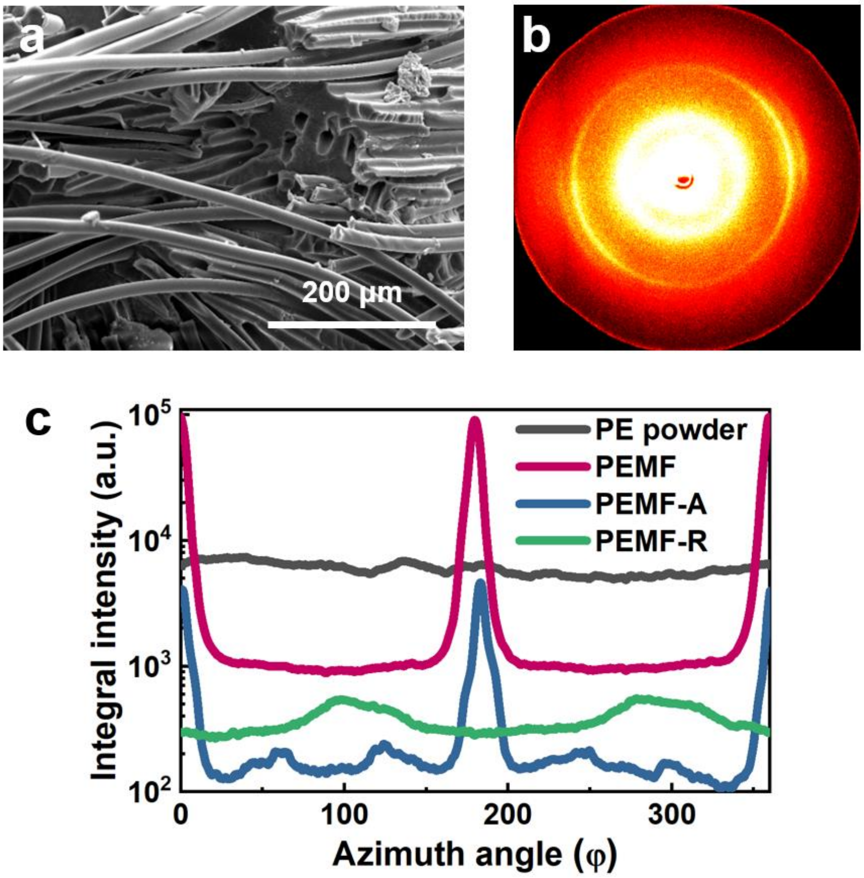

Figure S7. **Characterization of PEMF-R with the microfiber content of 55 wt%.** (a) SEM image and (b) its 2D WAXS image of PEMF-R showing the randomly distributed PEMF in PDMS matrix. (c) Integral intensity of (100) crystal vs azimuth angle curves of PE powder, PEMF and all PEMF-based materials.

WAXS analysis is another strong evidence for demonstrating this unique macroscopic morphology. According to 2D WAXS images of PEMF (Figure S5d), 2D WAXS image of PEMF-A material (Figure 1h) behave the similar two-point scattering patterns with intensive X-ray scattering only focused at bipolar in the meridional direction. In comparison, we also simply mixed PEMF into PDMS matrix to prepare the material with randomly distributed PEMF (PEMF-R material) (Figure S7a). Without this tactfully method, the arrangement of PEMF are highly disordered within the sample, and 2D WAXS image (Figure S7b) of PEMF-

R material with randomly distributed morphology only performs an isotropic characteristic. Meanwhile, the integral intensity vs azimuth angle curves (Figure S7c) of various PDMS/PEMF also demonstrate the fact that PEMF-A material possesses the highest degree of orientation, which is beneficial to construct highly oriented phonon-transporting in the vertical direction.

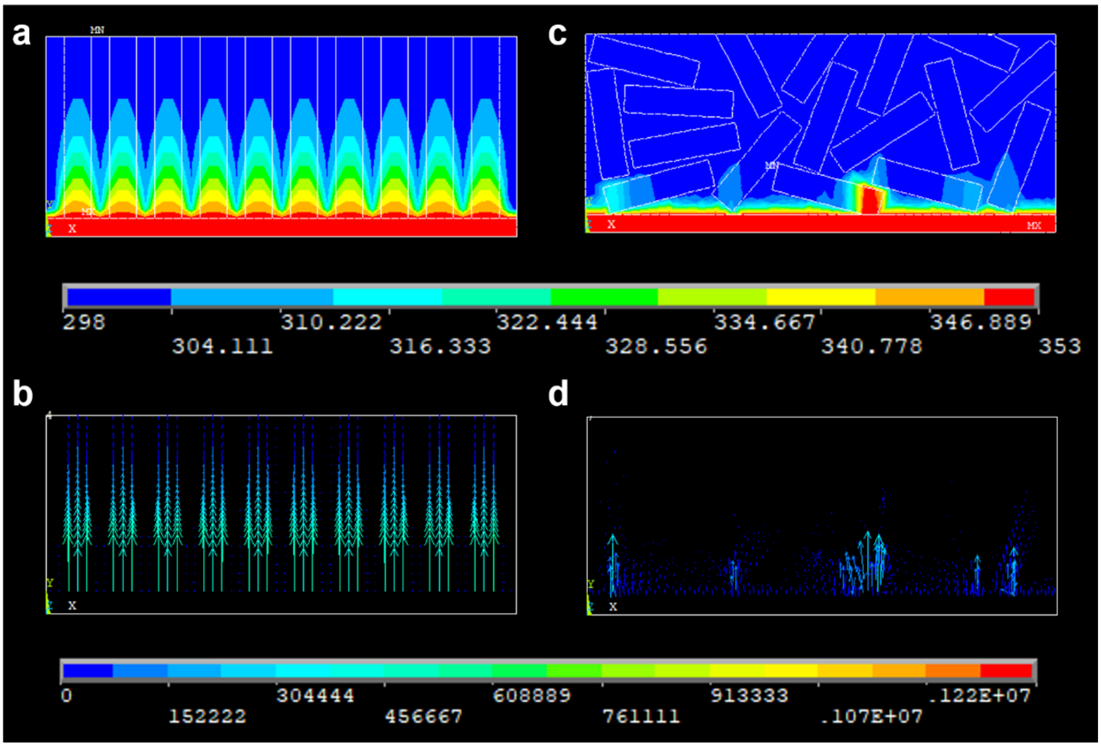

Figure S8. **Finite element analysis to visualize the heat-transfer process.** (a) temperature distribution and (b) heat flux at 2 s in PEMF-A material, and (c) temperature distribution and (d) heat flux at 2 s in PEMF-R material.

To investigate and visualize the circumstances of the heat transfer, a localized heat source (80 ^{o}C) with the diameter of 0.6 cm was applied on the bottom of the sample. The ambient temperature is set as 25 ^{o}C. During the heat dispassion process, the temperature and distribution of heat flux vector within composite are simulated based on classical Flourier Law $q= - k *grad(T)$, where q is the heat flux, k is the thermal conductivity, and $grad(T)$ is the temperature gradient, respectively.

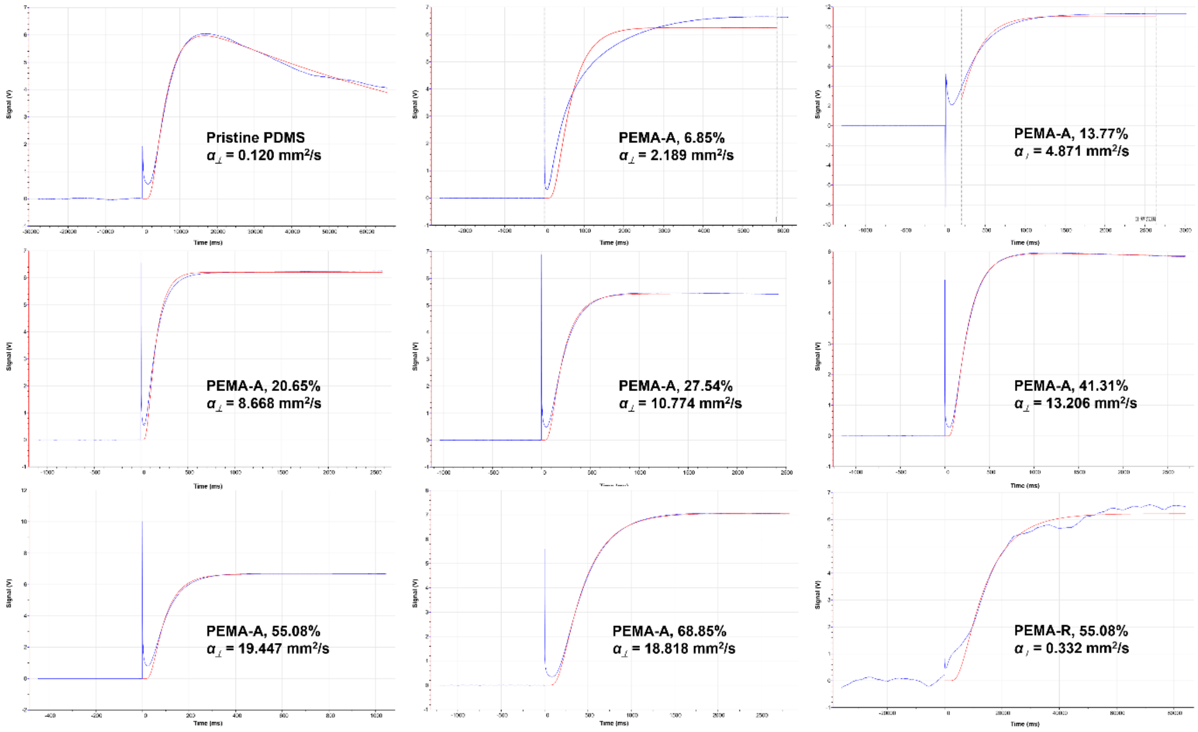

Figure S9. The raw data of LFA analysis. The raw data of LFA analysis for fitting the value of $\alpha_{\perp}$ of PDMS/PEMF material with different PEMF contents and PEMF orientations.

Table S1. Details for calculation of $\boldsymbol{\kappa}$ for PEMF-A materials at 30 ^{o}C.

Sample	0%	6.85%	13.77%	20.65%	27.54%	41.31%	55.08%	68.85%
C_p (J/g K)	1.78	1.794	1.808	1.821	1.835	1.863	1.890	1.918
ρ (g/cm^3)	1.2	1.178	1.156	1.135	1.115	1.077	1.041	1.008
$\alpha_{\perp}$ (mm^2/s)	0.116	2.348	4.775	8.769	10.752	14.735	19.451	18.675
$\kappa_{\perp}$ (W/m K)	0.248	4.962	9.980	18.124	21.999	29.565	38.270	36.105
$\alpha_{//}$ (mm^2/s)	0.116	0.122	0.175	0.198	0.199	0.23	0.24	0.272
$\kappa_{//}$ (W/m K)	0.248	0.258	0.366	0.409	0.407	0.461	0.472	0.526

ρ is the density that could be calculated from Equation 1, C_p is the specific heat capacity that could be calculated from Equation 2, and α is the thermal diffusion coefficient that could be calculated from Equation 3 as follows:

$$\rho_{PDMS/PEMF} = \frac{m_{PDMS}+m_{PEMF}}{\frac{m_{PDMS}}{\rho_{PDMS}}+\frac{m_{PEMF}}{\rho_{PEMF}}} \tag{1}$$

$$C_{p_{PDMS/PEMF}} = C_{p_{PDMS}} \frac{m_{PDMS}}{m_{PDMS}+m_{PEMF}} + C_{p_{PEMF}} \frac{m_{PEMF}}{m_{PDMS}+m_{PEMF}} \tag{2}$$

$$\alpha_{PDMS/PEMF} = \alpha_{PDMS} \frac{m_{PDMS}/\rho_{PDMS}}{\frac{m_{PDMS}}{\rho_{PDMS}}+\frac{m_{PEMF}}{\rho_{PEMF}}} + \alpha_{PEMF} \frac{m_{PEMF}/\rho_{PEMF}}{\frac{m_{PDMS}}{\rho_{PDMS}}+\frac{m_{PEMF}}{\rho_{PEMF}}} \tag{3}$$

Parallel Model analysis: PEMF are unidirectionally aligned within PDMS matrix in our study, and no PDMS-PEMF interface in the heat-transfer direction are introduced, therefore Parallel Model ($k_c = (1 - \varphi) * k_m + \varphi * k_a$) is very suitable for our system, where k_c, φ, k_m, and k_a represent thermal conductivity of polymer composites, volume fraction of additive, thermal conductivity of polymer matrix, and thermal conductivity of pristine additive, respectively. According to Parallel model, we have estimated the axial thermal conductivity of our PEMF to be ≈ 63 W/m K.

Table S2. **Comparison with previous literatures.** Comparison of *κ* of PEMF-A materials with other previously reported thermo-conductive but electrically insulating bulk materials.

Type	Matrix	Filler	Loading (wt%)	*κ* (W/m K)	*η* (%)	Ref.
Forest-like network	PDMS	PEMF	6.85	4.96	221	This work
	PDMS	PEMF	13.77	9.98	232	This work
	PDMS	PEMF	20.65	18.12	289	This work
	PDMS	PEMF	27.54	22.00	268	This work
	PDMS	PEMF	41.31	29.57	250	This work
	PDMS	PEMF	55.08	38.27	251	This work
3D interconnected network	Epoxy	BNNS	12.69	2.85	181	[1]
	Epoxy	BNNS	17.70	5.05	206	[2]
	Epoxy	BN	42.22	4.42	69.3	[3]
	Epoxy	BNNS	13.09	3.13	171	[4]
	PDMS	BNNS	28.7	1.94	55.77	[5]
	PLGA	BN	~ 54.76	2.1	23.75	[6]
	PVA	Al_2O_3	69.72	4.79	35.47	[7]
	Epoxy	BNNS	20.02	6.07	218	[8]
3D segregated network	PP	AlN	60.82	0.81	12.54	[9]
	PS	Si_3N_4	66.31	3	15.61	[10]
	PS	BNNS	25.07	0.58	18.00	[11]
	PS	BN/CNTs	23.97	0.67	26.26	[12]
	UHMWPE	BN/AlN	50	7.1	60.32	[13]
Field-induced orientation	PDMS	BN	15.99	~ 0.6	25.53	[14]
	HDPE	BN	40	3.57	~ 42.18	[15]
	Epoxy	BN	52.71	9	100	[16]
	RC	BN	30	2.97	~ 68.19	[17]
	UHMWPE	BN	70.50	12.42	6.28	[18]
	POE	BN	66.74	6.94	73.25	[19]
	PDMS	BN	21	1.5	42.61	[20]
	PDMS	BN	44.77	5.47	118	[21]
Other methods	Epoxy	BNNT	30	2.77	42.83	[22]
	Epoxy	Al_2O_3	84	13.46	105.4	[23]
	Epoxy	BNNS/AgNPs	32.22	3.06	44.79	[24]
	Epoxy	BN/GO	30	0.94	17.01	[25]

PP- polyproplene, BN- hexagonal boron nitride, BNNS- boron nitride nanosheets, HDPE- high density polyethylene, PLGA- poly(lactic-co-glycolic acid), RC- regenerated cellulose, PS- polystyrene, PVA- poly(vinyl alcohol), UHMWPE- ultrahigh molecular weight polyethylene, POE- polyolefin elastomer, SiCNW- SiC nanowire, BNNT- boron nitride

nanotubes, AgNPs- silver nanoparticles, CNTs- carbon nanotubes, GO- graphene oxide, and η is calculated according to the Equation 4 as follows:

$$\eta = \frac{k - k_m}{100 v_f * k_m} \tag{4}$$

where κ and κ_m are the thermal conductivity of polymer composite and polymer matrix, and V_f is the filler volume fraction.

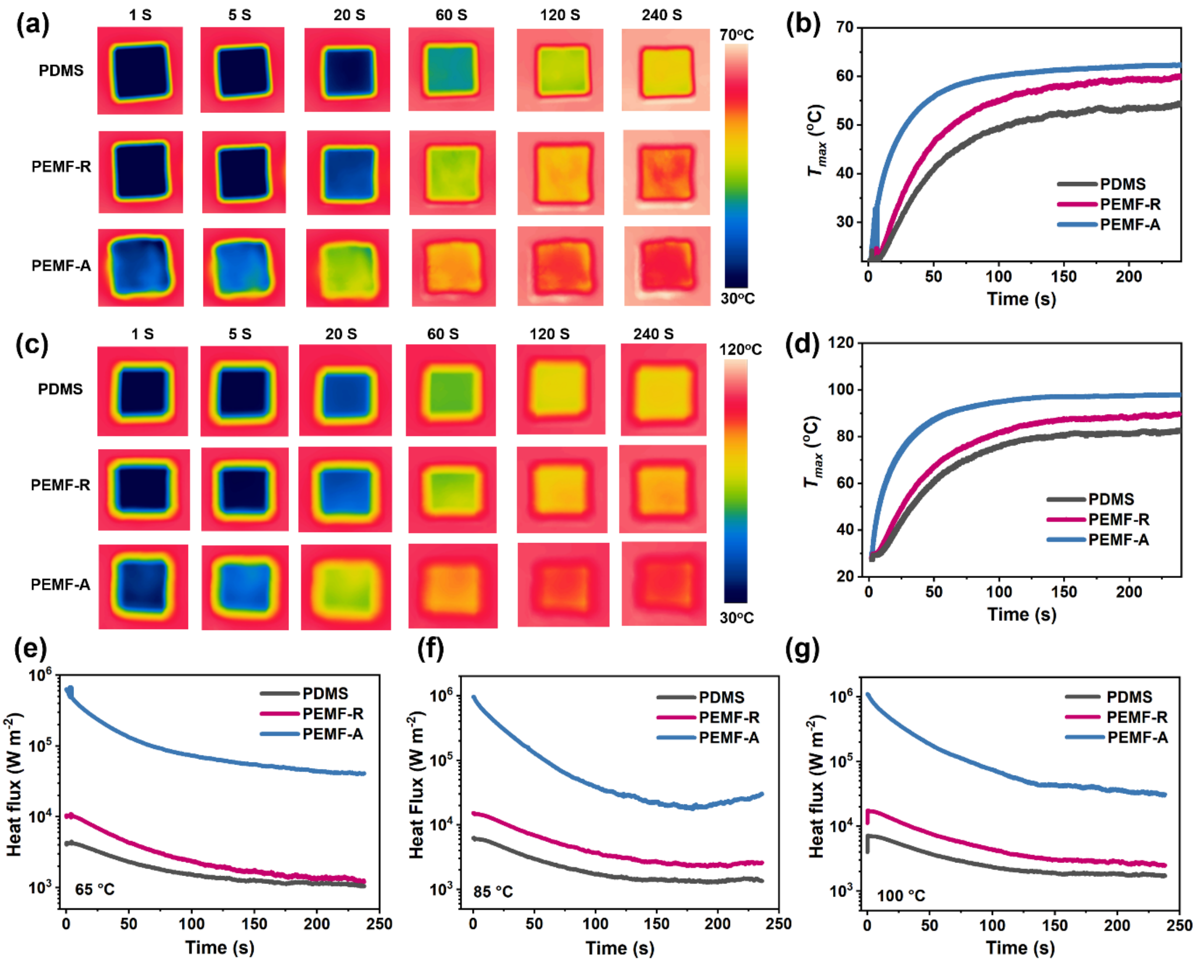

Figure S10. Infrared camera recorded results. (a) Infrared images and (b) recorded temperatures in PDMS, PEMF-R and PEMF-A as they are put on a hot plate set at 65 ^{o}C; (c) Infrared images and (d) recorded temperatures in PDMS, PEMF-R and PEMF-A as they are put on a hot plate set at 100 ^{o}C; Heat flux within TIM of pristine PDMS, PEMF-R and PEMF-A at different times, as they are put on a hot plate set at (e) 65 ^{o}C, (f) 85 ^{o}C, (g) 100 ^{o}C, respectively.

Noted that the heat flux provided in Figure S10 was calculated based on the power input of hot plate, while ignoring the influence of heat convention and heat radiation with surroundings.

5.3 Analysis of interfacial thermal resistance

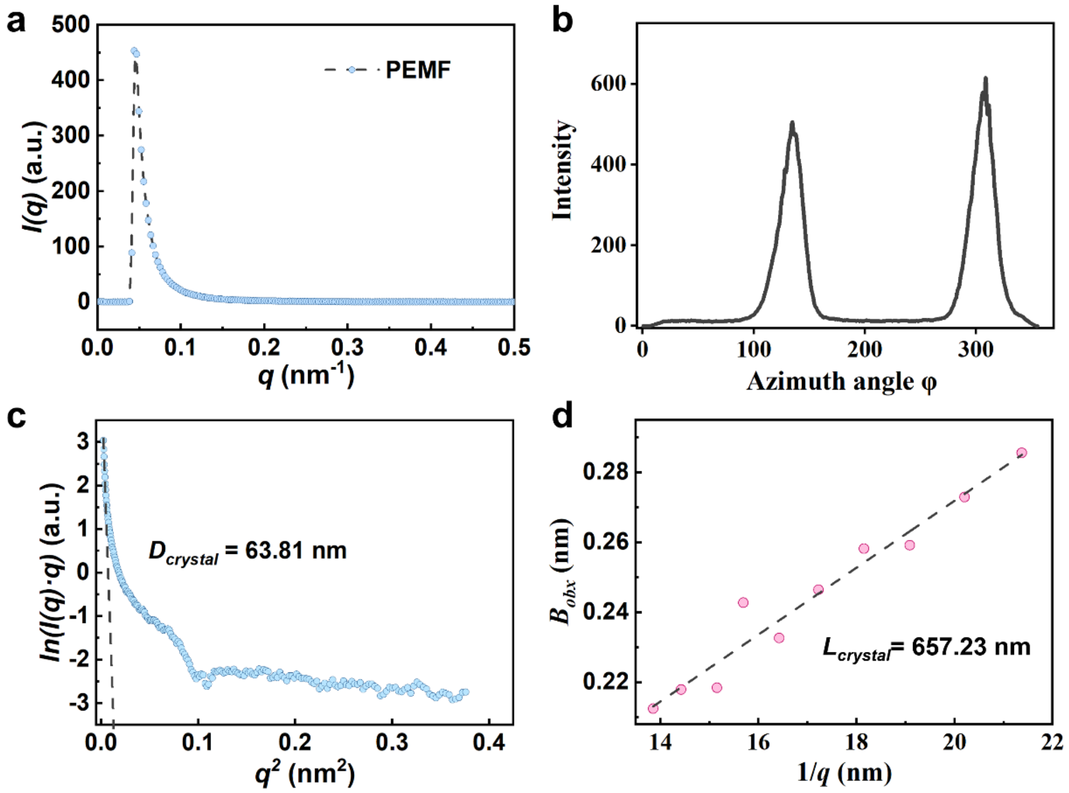

Figure S11. **Analysis of PEMF's crystalline structure.** (a) The fitting intensity (I (q)) curves of PEMF along the direction of the scattering wave vector (q). (b) The fitting I (ϕ) curves of PEMF, in which ϕ means the azimuth angle. (c) The fitting curves of Guniner approximation expansion of PEMF. (d) The fitting curves of the integral width of one-dimensional azimuth at different q values along the direction of equator (B_{obs}) and $1/q$ of PEMF.

With the help of 2D SAXS analysis and Fit 2D software packaging, we could obtain detailed structural information of PEMF samples, such as the curves of I (q), average diameter of shish crystals of PEMF and average length of shish crystals of PEMF (Figure S11).

Curves of I (q) and I (ϕ): The 2D SAXS analysis is often related to the scattering wave vector q (nm^{-1}) and the scattering intensity I. As for the curves of I (q), with the help of Fit 2D

software, we could integrate the patterns by meridian scan along the direction of q, in order to gain the results of scattering intensity distribution. Figure S11a shows the $I(q)$ curve of PEMF, which clarifies the existence of long, oriented fibrillar, shish crystals.

Meanwhile, the curves of the scattering intensity I and the azimuth angle ϕ could also be obtained. As for the curves of $I\ (\phi)$, with the help of Fit 2D software, we could integrate the patterns with the whole range of azimuth angle ϕ (0-360º). Besides the obvious 2D SAXS scattering patterns of sample, the $I\ (\phi)$ curve of PEMF obviously shows the double peaks in Figure S11b, which gives a great assistance to manifest the highly oriented structures of PEMF.

Calculation of average diameters of shish crystals of PEMF: From the previous research about shish-kebab structure, the specific shish structure could be considered as a collection of long and smooth extended-chain crystals, like a stick.[26] Thus, according to the "Guinier approximation",[27] the average diameters of fibrillar shish crystal ($D_{crystal}$) could be gained by Equation 5 as follows:

$$I \propto \frac{e^{\frac{-{R_g}^2 q^2}{2}}}{q} \qquad (5)$$

According to scattering pattern of 2D SAXS, the Guniner approximation expansion could be gained by Equation 6:

$$\ln(I(q) \cdot q) = \frac{-{R_g}^2}{2} \cdot q^2 + lnI_0 \qquad (6)$$

R_g is the radius of gyration of rod-like crystals, $I(q)$ and q could be attained by the above curves of $I\ (q)$. At the same time, the shish structure is regarded as symmetrical cylinder, so the $D_{crystal}$ could be calculated as follows:

$$D_{crystal} = 8^{1/2} R_g \qquad (7)$$

From the fitting curve of Guniner approximation expansion, there are two parts of the fitting curves. In the first part (q < 0.015nm), the mentioned Equation 6 and 7 could be used to calculate the $D_{crystal}$. The slope of Guniner approximation expansion could be easily gained and the R_g, $D_{crystal}$ could also be attained. The fitting curve of Guniner approximation expansion of PEMF is in Figure S11c.

Calculation of average length of shish crystals of PEMF: According to Peter and Ruland method,[28] the average length of fibrillar shish crystals ($L_{crystal}$) of PEMF could be obtained. In this method, the B_{obs} means the integral width of one-dimensional azimuth at different q values along the direction of equator. The B_{obs} could be calculated as follows:

$$B_{obs}(q) = \frac{1}{I(q,\pi/2)} \int_{-\pi/2}^{\pi/2} I(q,\phi) d\phi \qquad (8)$$

ϕ is the azimuth angle; $I\ (q,\ \phi)$ is the scattering intensity. If all the azimuthal distribution could be modeled by Lorentz functions, the relation of B_{obs} and $L_{crystal}$ is as follows:

$$B_{obs} = b_{\phi} + 2\pi/(L_{crystal} \cdot q) \qquad (9)$$

b_{ϕ} is the misorientation factors of shish. If the azimuthal distribution accord with Gaussian expressions, then the relation of B_{obs} and $L_{crystal}$ is as follows:

$$B_{obs}{}^{2} = b_{\phi}{}^{2} + 4\pi^{2}/(L_{crystal} \cdot q)^{2} \qquad (10)$$

$L_{crystal}$ could be obtained from the slope of the equations. In this study, all azimuthal distributions were found to be better fit with Lorentz functions, thus the Equation 9 was determined to calculate the $L_{crystal}$. The fitting curves of B_{obs} and $1/q$ is shown in Figure S11d.

Modified EMT model analysis: $R_{crystal\text{-}amorphous}$ is quantified according to the modified EMT model[29], in which PDMS with amorphous region of PEMF is taken as the matrix, while the crystalline region of PEMF is considered as the additive. Its value is fitted according to Equation 11 as follows:

$$\frac{\kappa_e}{\kappa_m} = 1 + \frac{fp}{3}\frac{\kappa_c/\kappa_m}{p+\frac{2R_{crystal-amorphous}\kappa_m}{D}\frac{\kappa_c}{\kappa_m}} \qquad (11)$$

where κ_e is the κ of PEMF-A materials, κ_m is the κ of PDMS/amorphous PEMF matrix, f is the volume content of fibrillar nanocrystal, p is the aspect ratio, D is the diameter of fibrillar nanocrystal. Herein, D and p were respectively measured by SAXS (Figure S11). It was reported that an individual PE fibrillar nanocrystals could achieve an axial κ of 237 W/m K, in consideration of the crystalline size based on diffuse phonon scattering at boundaries.[30] Series Model was used to quantify the thermal conductivity of amorphous region of PEMF (30.89 W/m K) according to this literature (Equation 12),[31] as well as the κ_m (Equation 13). Based on these parameters, $R_{crystal-amorphous}$ is estimated to be 7.77*10^{-9} m^2 K W^{-1}.

$$\kappa_f = (\frac{f}{\kappa_c} + \frac{1-f}{\kappa_a})^{-1} \qquad (12)$$

where f is the volume content of fibrillar nanocrystal in PEMF, κ_f, κ_c, and κ_a are the thermal conductivity of PEMF, fibrillar nanocrystal of PEMF, and amorphous region of PEMF, respectively.

$$\kappa_m = (\frac{\varphi}{\kappa_a} + \frac{1-\varphi}{\kappa_p})^{-1} \qquad (13)$$

where φ is the volume fraction of amorphous region of PEMF in PDMF/PEMF, κ_m, κ_a, and κ_p are the thermal conductivity of PDMS/amorphous PEMF matrix, amorphous region of PEMF and PDMS, respectively.

Nonlinear model (Foygel et al.) analysis: The nonlinear model proposed by Foygel et al.[32] is used to fit the contact thermal resistance ($R_{crystal-crystal}$) (Figure 3b), where it is noteworthy that PDMS with amorphous region of PEMF is taken as the matrix, while the crystalline region of PEMF is considered as the additive. The equations are provided as Equation 14 and Equation 15 as follows:

$$\kappa_e = \kappa_0(V_f - V_c)^{\tau} \qquad (14)$$

$$R_{crystal-crystal} = \frac{\pi \mathrm{DLS}}{\kappa_0 L (V_c)^{\tau}} \quad (15)$$

where κ_0 is a preexponential parameter that is the estimated contribution of fibrillar nanocrystal network alone, V_f is the volume percentage of fibrillar nanocrystal, V_c is the critical volume percentage of fibrillar nanocrystal, L is the length of fibrillar nanocrystal, S is the average contact area between adjacent two crystals, and τ is a conductivity exponent that is determined by the aspect ratio of fibrillar nanocrystal. Considering usually limited contact area from fibrous crystals due to the restricted linear overlap, thus <1/1000 of fibrillar crystal's surface area is assumed as S. Based on this, $R_{crystal-crystal}$ is determined to be ≈$4.1*10^{-11}$ m^2 K W^{-1}. This exceedingly low interfacial thermal resistance attributes to the contribution of amorphous regions of PEMF that have bridged adjacent fibrillar nanocrystals within the PDMS/PEMF material.

5.4 Additional performance of PEMF-A required in thermal managements

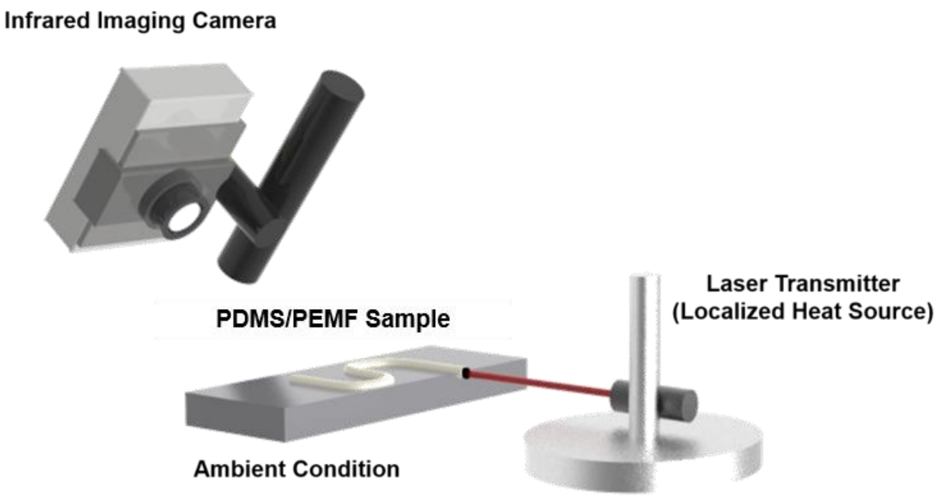

Figure S12. **Schematic illustration of heating process of PEMF-A with "S" shape.** In virtue of laser transmitter (heat source, 2.8 W) and infrared imaging camera, the thermo-conductive performances could be obviously exhibited.

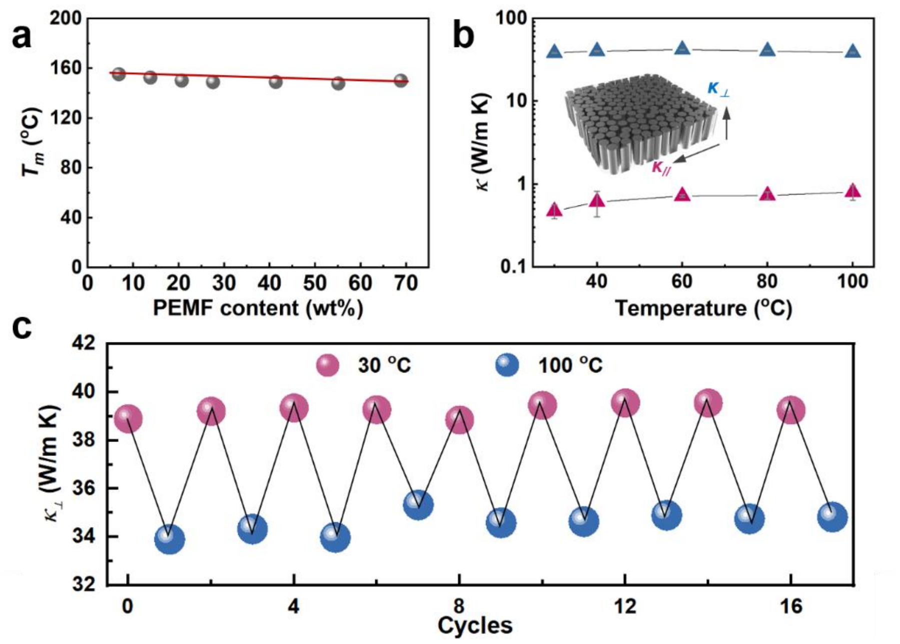

Figure S13. **Thermal stability of PEMF-A material.** (a) DSC defined T_m of PEMF-A material as a function of PEMF content. (b) Anisotropic thermal conductivities of PEMF-A material at different temperatures. (c) Typical $\kappa_{\perp}$ of PEMF-A material after repeated heating and cooling cycles.

To explore the thermal stability, anisotropic thermal conductivities at different temperatures were studied. Firstly, DSC analysis was used to confirm the critical melting points (T_m) of PEMF-A materials, which manifests a stable T_m around 150 ^{o}C (Figure S13a). Figure S13b and Figure S13c further verify this conclusion, as both anisotropic thermal conductivities of PEMF-A material behave a gradual variation as increase of the test temperature, and the small decrease in $\kappa_{\perp}$, derived from the increase of specific heat capacity but the decrease of thermal diffusion coefficient (Figure S14, Table S3 and Table S4). All of parameters could be in the stable state after 17 times of heating or cooling cycles. These excellent thermo-conductive characteristic at variable temperatures is ascribed to the highly efficient phonon highways, in which fibrillar nanocrystals, crystal-amorphous interfaces and crystal-crystal contacts could be durable at higher temperatures.

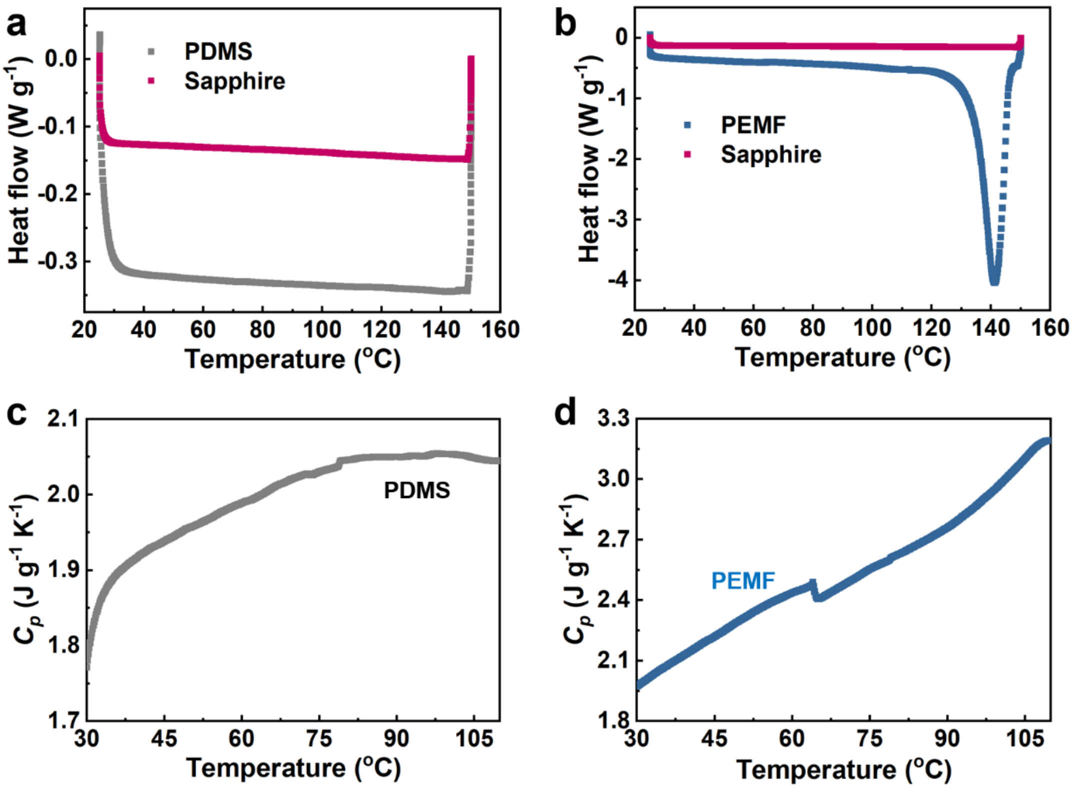

Figure S14. **DSC analysis for confirm the C_p of PDMS and PEMF.** DSC curves of (a) PDMS and (b) PEMF as function of the temperature. Calculated C_p of (c) PDMS and (d) PEMF at the different temperatures.

Table S3. C_p of PEMF, PDMS, and PEMF-A material at different temperatures. Noted that the filling ratio of PEMF-A is 55 wt%.

**Temperature (^oC)**	**30**	**40**	**60**	**80**	**100**
PEMF	1.98	2.14	2.44	2.62	2.97
PDMS	1.78	1.92	1.99	2.04	2.05
PEMF-A	1.890	2.041	2.238	2.359	2.557

Table S4. The anisotropic α of PEMF-A material with 55 wt% PEMF at different temperatures.

Temperature (^{o}C)	30	40	60	80	100
$\alpha_{//}$ (mm^2/s)	0.472	0.612	0.72	0.732	0.801
$\alpha_{\perp}$ (mm^2/s)	19.451	18.94	17.983	16.42	14.477

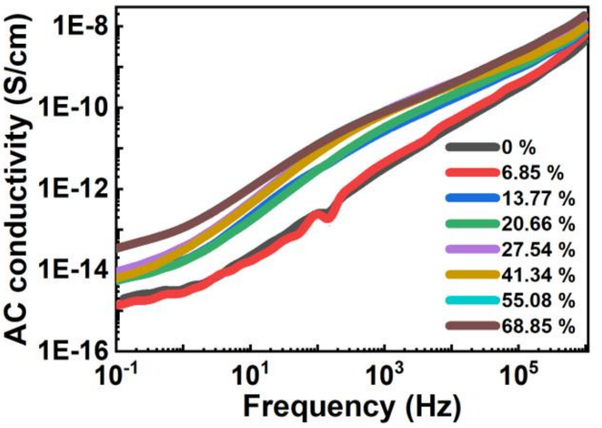

Figure S15. **Electrical insulating properties of PEMF-A materials.** The altering current (AC) conductivity of PEMF-A material as increase of the frequency.

Figure S15 plots the AC conductivity as a function of the frequency. Although the introduction of PEMF leads to a little increase of the AC electrical conductivity attributed to the inevitable interfacial polarizations at PDMS-PEMF interfaces, PEMF-A material with 55 wt% PEMF could still maintain excellent electrical insulation ($3.46*10^{-14}$-$1.87*10^{-8}$ S/cm, 0.1-10^6 Hz) even at 10^6 Hz, which successfully demonstrate its impressive electrical insulating properties for practical applications.

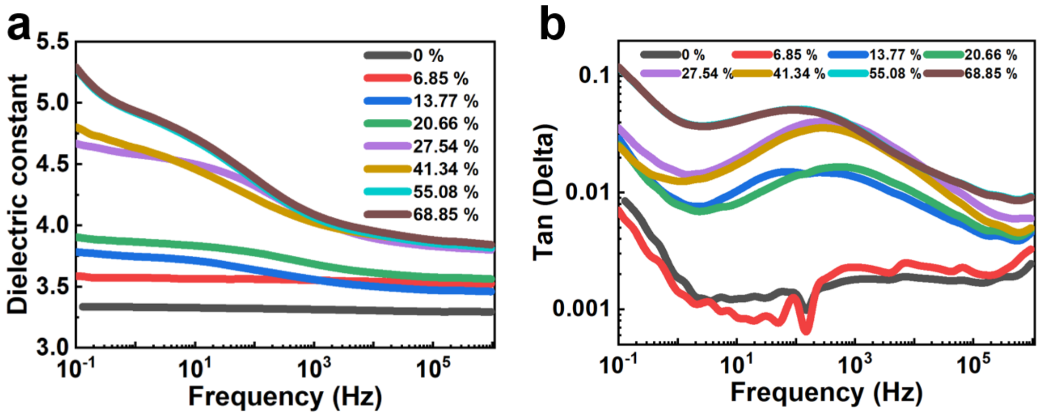

Figure S16. **Dielectric analysis of PEMF-A materials.** (a) Dielectric constant, and (b) dielectric loss of PEMF-A material as increase of the frequency.

5.5 COB device performance

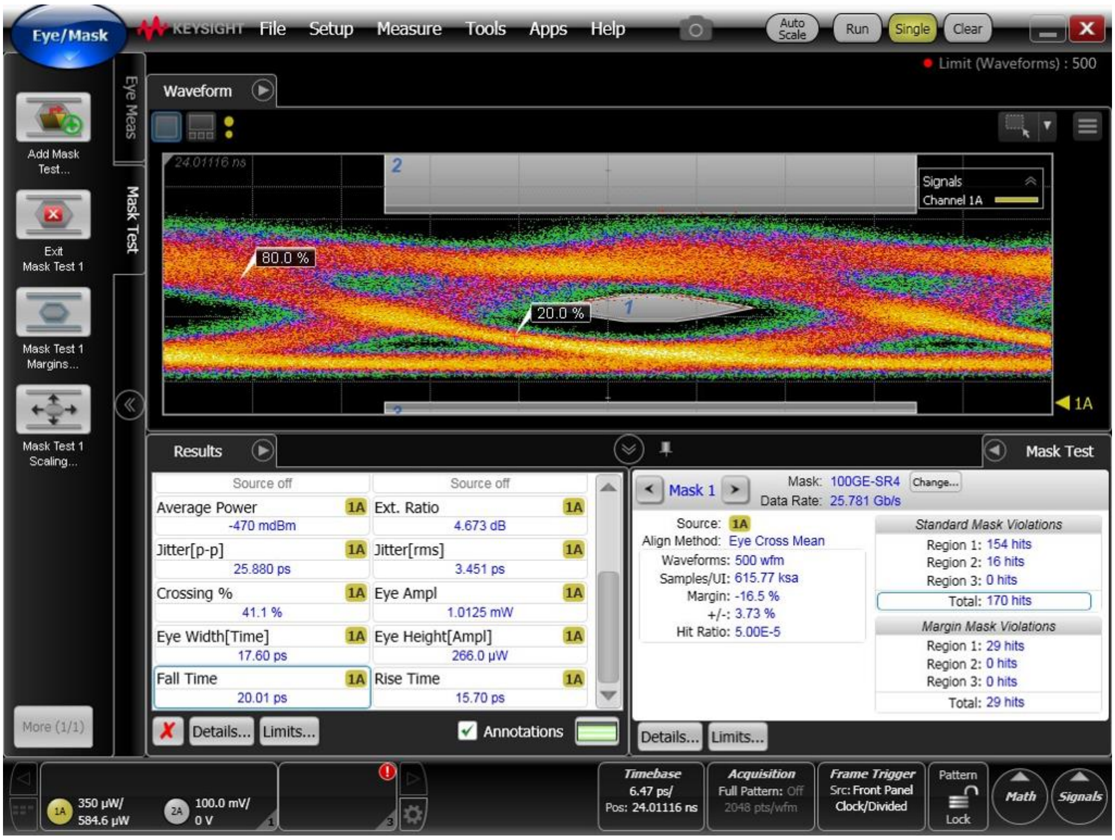

Figure S17. **COB packed chips without TIM at the board-heat sink interface.** The details of eye diagram for COB packed chip with air at the board-heat sink interface.

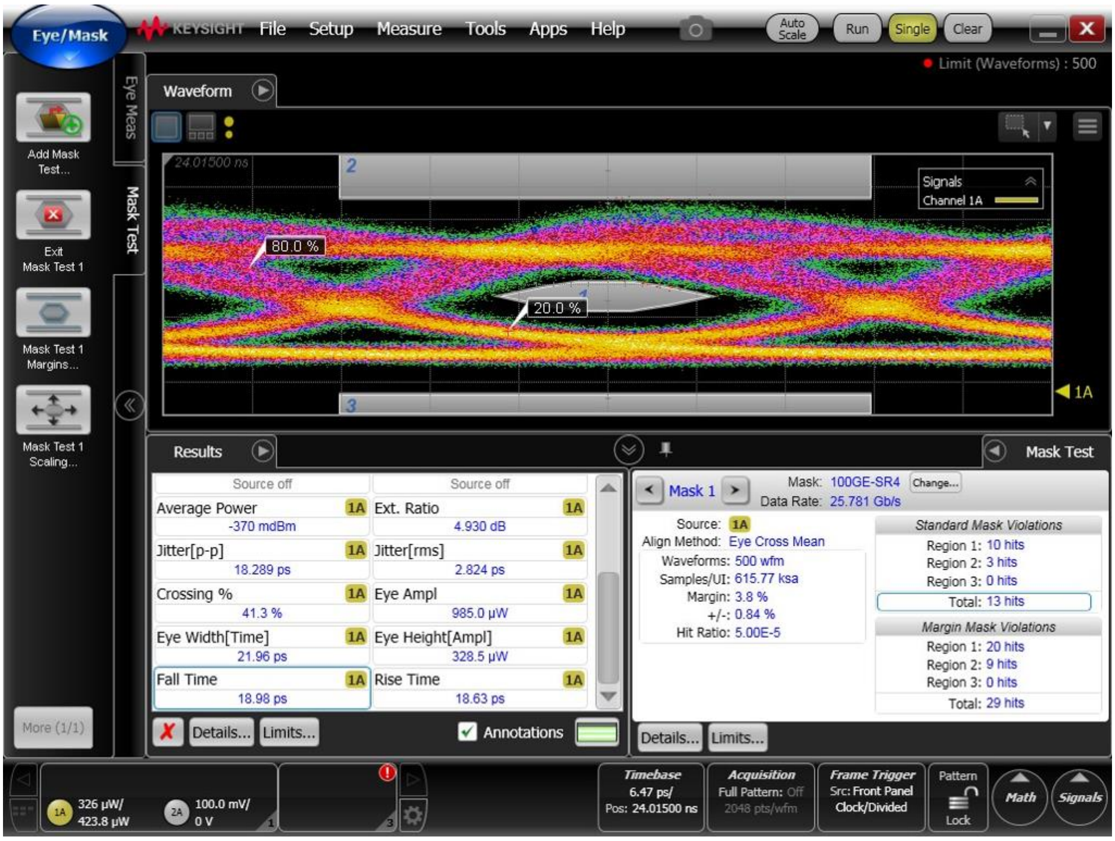

Figure S18. **COB packed chips with pristine PDMS as TIM at the board-heat sink interface.** The details of eye diagram for COB packed chip with pristine PDMS at the board-heat sink interface.

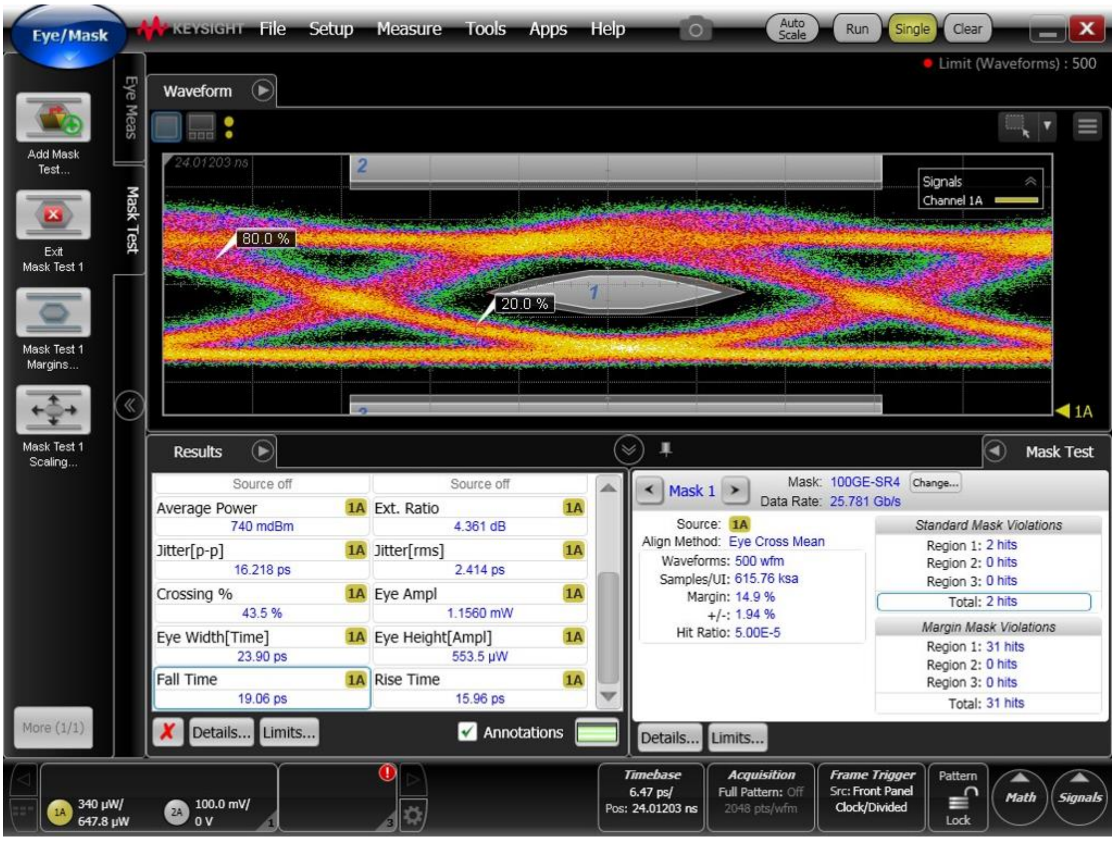

Figure S19. **COB packed chips with PEMF-A material as TIM at the board-heat sink interface.** The details of eye diagram for COB packed chip with PEMF-A at the board-heat sink interface.

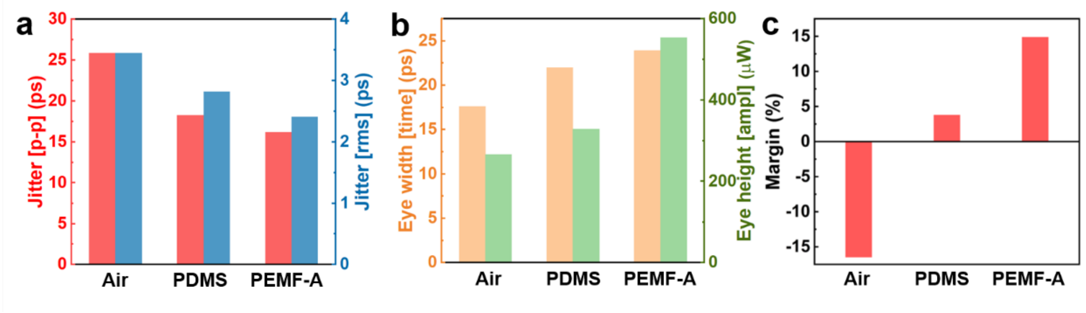

Figure S20. **Working parameters of a COB technique packed chip.** Working parameters, including (a) jitter, (b) eye dimensions, and (c) margin of the COB technique packed chip using air, PDMS and PEMF-A as the TIMs, respectively.

The eye diagram could directly and rapidly check the transmission quality of digital signal. From the parameters of eye diagrams, the working situations of chips or LED devices could be efficiently analyzed. Due to amount of superposition of signals, jitter could be clearly shown in the time horizontal axis of eye diagram. The distance of vacuum area in the horizontal axis were named the eye width, which could be better reflect stability of signal transmission. Meanwhile, the distance of vacuum area in the vertical axis were named as eye height, which could be better reflect noise margin of signal transmission. In virtue of combination with COB packed chips, our material with metal-like κ could directly exhibit a promising potential in the giant power devices in Figure S19.

Detailly, the working parameters, including node temperature, jitter, eye dimensions, and margin obtained from the eye diagram analysis were respectively studied at room temperature. According to the eye diagrams, COB chip packed with our PEMF-A materials performs the lowest jitter in the time domain that reflects the excellent signal stability as shown in Figure S20a. In addition, the eye dimensions (Figure S20b) including eye width and eye height also indicates our PEVF-A supported COB chip possesses the largest margin (Figure S20c) for signal transmission, while that of air or pristine PDMS packed with COB chip has been seriously obstructed; this highlights the significance of heat-dissipation in chips' packaging

and one more time manifests the exquisite thermo-conductive characteristic of our engineered PEMF-A organics.

5.6 Mechanical properties

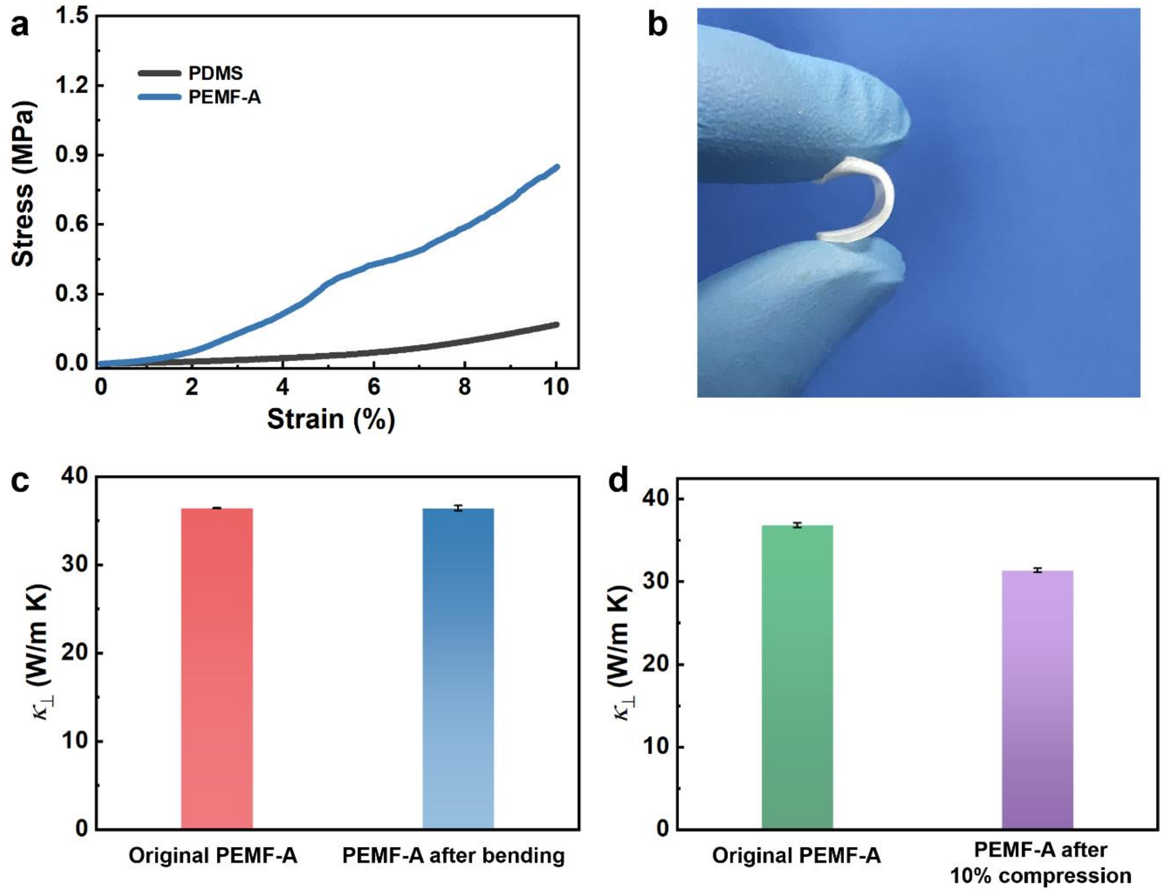

Figure S21. Mechanical properties. (a) Compressive stress-strain curves of pristine PDMS and PEMF-A with 55% PEMF content; (b) Photograph demonstrates PEMF-A bulk material with thickness of 1 mm could be bendable. (c) The characterized $\kappa_\perp$ of PEMF-A before and after bending or (d) 10% compression.

As shown in Figure S21a (Supporting Information), PEMF-A material with 55 wt% PEMF could behave retained compressive properties, including relatively low modulus (1.658±0.332 MPa) and compression strength (840±58 kPa). Pristine PDMS material exhibits modulus of (0.500±0.585 MPa) and compression strength of (196±27 kPa). Moreover, images in Figure S21b (Supporting Information) demonstrates the ability of PEMF-A to be bended without any breakage, indicating its good mechanical properties. In Figure S21c and S21d, we also

provided the $\kappa_{\perp}$ of PEMF-A before and after bending or compression. It was found that bending procedure give rise to no obvious degradation of $\kappa_{\perp}$, while after 10% compression, the $\kappa_{\perp}$ of PEMF-A has decreased by 14.8%. We think that vertically aligned PEMF bunches would be bended during the compression procedure, while they could not revert to the original configuration although the PDMS matrix could.

Author contributions

K.W. designed the experiments. K.W. and Q.F. supervise the experiments. Y.Z.Z. fabricated the materials. K.W., Y.Z.Z. and C.X.L. performed the experimental tests. K.W. and Q.F. analyzed the data. K.W. and Q.F. wrote the paper.

References

[1] X. Zeng, Y. Yao, Z. Gong, F. Wang, R. Sun, J. Xu, C. P. Wong, *Small* **2015**, 11, 6205.
[2] Y. Yao, J. Sun, X. Zeng, R. Sun, J. B. Xu, C. P. Wong, *Small* **2018**, 14, 1704044.
[3] J. Hu, Y. Huang, Y. Yao, G. Pan, J. Sun, X. Zeng, R. Sun, J.-B. Xu, B. Song, C.-P. Wong, *ACS Appl. Mater. Interfaces* **2017**, 9, 13544.
[4] J. Chen, X. Huang, Y. Zhu, P. Jiang, *Adv. Funct. Mater.* **2017**, 27, 1604754.
[5] J. Chen, X. Huang, B. Sun, Y. Wang, Y. Zhu, P. Jiang, *ACS Appl. Mater. Interfaces* **2017**, 9, 30909.
[6] L. M. Guiney, N. D. Mansukhani, A. E. Jakus, S. G. Wallace, R. N. Shah, M. C. Hersam, *Nano Lett.* **2018**, 18, 3488.
[7] Y. Wu, K. Ye, Z. Liu, M. Wang, K. W. Chee, C.-T. Lin, N. Jiang, J. Yu, *J. Mater. Chem. C* **2018**, 6, 6494.
[8] J. Han, G. Du, W. Gao, H. Bai, *Adv. Funct. Mater.* **2019**, 29, 1900412.
[9] M. Hu, J. Feng, K. M. Ng, *Compos. Sci. Technol.* **2015**, 110, 26.
[10] H. He, R. Fu, Y. Shen, Y. Han, X. Song, *Compos. Sci. Technol.* **2007**, 67, 2493.
[11] X. Wang, P. Wu, *ACS Appl. Mater. Interfaces* **2017**, 9, 19934.
[12] K. Wu, Y. Li, R. Huang, S. Chai, F. Chen, Q. Fu, *Compos. Sci. Technol.* **2017**, 151, 193.
[13] Z.-G. Wang, F. Gong, W.-C. Yu, Y.-F. Huang, L. Zhu, J. Lei, J.-Z. Xu, Z.-M. Li, *Compos. Sci. Technol.* **2018**, 162, 7.
[14] C. Yuan, B. Duan, L. Li, B. Xie, M. Huang, X. Luo, *ACS Appl. Mater. Interfaces* **2015**, 7, 13000.
[15] X. Zhang, J. Zhang, L. Xia, C. Li, J. Wang, F. Xu, X. Zhang, H. Wu, S. Guo, *ACS Appl. Mater. Interfaces* **2017**, 9, 22977.
[16] C. Yu, J. Zhang, Z. Li, W. Tian, L. Wang, J. Luo, Q. Li, X. Fan, Y. Yao, *Composites, Part A* **2017**, 98, 25.
[17] J. Lao, H. Xie, Z. Shi, G. Li, B. Li, G.-H. Hu, Q. Yang, C. Xiong, *ACS Sustainable Chem. Eng.* **2018**, 6, 7151.
[18] Y.-F. Huang, Z.-G. Wang, H.-M. Yin, J.-Z. Xu, Y. Chen, J. Lei, L. Zhu, F. Gong, Z.-M. Li, *ACS Appl. Nano Mater.* **2018**, 1, 3312.
[19] C.-P. Feng, L. Bai, R.-Y. Bao, Z.-Y. Liu, M.-B. Yang, J. Chen, W. Yang, *Adv. Compos. Hybrid Mater.* **2018**, 1, 160.
[20] Z. Liang, Y. Pei, C. Chen, B. Jiang, Y. Yao, H. Xie, M. Jiao, G. Chen, T. Li, B. Yang, *ACS Nano* **2019**, 13, 12653.
[21] Z. Kuang, Y. Chen, Y. Lu, L. Liu, S. Hu, S. Wen, Y. Mao, L. Zhang, *Small* **2015**, 11, 1655.
[22] X. Huang, C. Zhi, P. Jiang, D. Golberg, Y. Bando, T. Tanaka, *Adv. Funct. Mater.* **2013**, 23, 1824.
[23] Y. Hu, G. Du, N. Chen, *Compos. Sci. Technol.* **2016**, 124, 36.
[24] F. Wang, X. Zeng, Y. Yao, R. Sun, J. Xu, C.-P. Wong, *Sci. Rep.* **2016**, 6, 1.

[25] K. Wu, C. Lei, W. Yang, S. Chai, F. Chen, Q. Fu, *Compos. Sci. Technol.* **2016**, 134, 191.

[26] A. Pennings, J. Van der Mark, A. Kiel, *Kolloid-Zeitschrift und Zeitschrift für Polymere* **1970**, 237, 336.

[27] a) A. W. Phillips, A. Bhatia, P.-w. Zhu, G. Edward, *Macromolecules* **2011**, 44, 3517; b) J. K. Keum, F. Zuo, B. S. Hsiao, *Macromolecules* **2008**, 41, 4766.

[28] R. Perret, W. Ruland, *J. Appl. Crystallogr.* **1969**, 2, 209.

[29] a) C.-W. Nan, R. Birringer, D. R. Clarke, H. Gleiter, *J. Appl. Phys.* **1997**, 81, 6692; b) K. Wu, Y. Xue, W. Yang, S. Chai, F. Chen, Q. Fu, *Compos. Sci. Technol.* **2016**, 130, 28.

[30] X. Wang, M. Kaviany, B. Huang, *Nanoscale* **2017**, 9, 18022.

[31] Y. Xu, D. Kraemer, B. Song, Z. Jiang, J. Zhou, J. Loomis, J. Wang, M. Li, H. Ghasemi, X. Huang, *Nat. Commun.* **2019**, 10, 1.

[32] M. Foygel, R. Morris, D. Anez, S. French, V. Sobolev, *Phys. Rev. B* **2005**, 71, 104201.
